# Heparin in sepsis: current clinical findings and possible mechanisms

**DOI:** 10.3389/fimmu.2024.1495260

**Published:** 2024-12-06

**Authors:** Sihan Yu, Yawen Chi, Xiaochun Ma, Xu Li

**Affiliations:** Department of Critical Care Medicine, The First Affiliated Hospital of China Medical University, Shenyang, Liaoning, China

**Keywords:** sepsis, coagulation, heparin, anticoagulant, mechanism

## Abstract

Sepsis is a clinical syndrome resulting from the interaction between coagulation, inflammation, immunity and other systems. Coagulation activation is an initial factor for sepsis to develop into multiple organ dysfunction. Therefore, anticoagulant therapy may be beneficial for sepsis patients. Heparin possesses a variety of biological activities, so it has a broad prospect in sepsis. Previous studies suggested that patients with sepsis-induced disseminated intravascular coagulation and high disease severity might be suitable for anticoagulant therapy. With the development of artificial intelligence (AI), recent studies have shown that patients with severe coagulation activation represent the targeted patients for anticoagulant therapy in sepsis. However, it remains necessary to accurately define the relevant biomarkers indicative of this phenotype and validate their clinical utility by large randomized controlled trials (RCTs). Analyses of data from early small RCTs, subgroup analyses of large RCTs and meta-analyses have collectively suggested that anticoagulant therapy, particularly the use of heparin, may be an effective approach for managing sepsis patients. Concurrently, debate persists regarding the optimal selection of anticoagulants, proper timing, usage and dosage of administration that should be employed to assess treatment efficacy. The primary mechanisms of heparin are acting on heparan sulfate, histones, high mobility group box 1 and heparin-binding protein, which interfere with the regulation of inflammation, vascular permeability, coagulation, endothelial function and other biological activities. However, the underlying pathophysiological processes mediating the potential therapeutic effects of heparin in the context of sepsis remain incompletely understood and warrant additional rigorous investigation to establish the mechanism more conclusively.

## 1 Introduction

Sepsis 3.0 puts more emphasis on the ultimate impacts of infection on the patients (1). In the early stages of sepsis, the inflammatory response initiates and promotes coagulation activation, leading to microvascular thrombosis. The complex interaction between inflammation, immune response and coagulation promotes the progression of sepsis, and leads to widespread thrombosis, which reduces tissue perfusion and leads to organ dysfunction. Therefore, coagulation dysfunction is considered to be an initial factor in the progression of sepsis to multiple organ dysfunction syndrome (MODS) (2). Endothelial cells, which line the luminal surface of all blood vessels, are the first barrier separating blood from organs (3). Therefore, endothelial cells are the first sites to be invaded by pathogens. In sepsis, damaged endothelial cells interact with activated clotting factors and platelets, leading to thrombosis and tissue hypoperfusion (4, 5). Therefore, anticoagulant therapy may be beneficial. Many studies have investigated the effects of various anticoagulants on sepsis patients, but no positive results are reported to date. Heparin, as a commonly used anticoagulant in clinical practice, also has unique biological activities such as anti-inflammatory, immunomodulatory, and vascular-protective effects. These non-anticoagulant effects may also play an important role in improving the prognosis of sepsis. This review focuses on the role of heparin in sepsis, including the results of recent clinical studies and related mechanisms.

## 2 Necessity of anticoagulant therapy in sepsis

In 2016, the definition of sepsis was updated to sepsis 3.0, which refers to “life-threatening organ dysfunction caused by a dysregulated host response to infection” (1). This represents a significant advancement in the understanding of the pathophysiology underlying the development and progression of sepsis, with more emphasis on the ultimate systemic impacts resulting from the initial infection. In sepsis, pathogens invade the body and induce the production of proinflammatory cytokines, which further damage endothelial cells and promote coagulation activation. The interaction of inflammation and coagulation regulates microvascular thrombosis (6). In fact, almost all sepsis patients develop abnormal coagulation function (7). These abnormalities range from mild coagulation activation that can only be detected by sensitive biomarkers to fulminant disseminated intravascular coagulation (DIC). The three major factors involved in the coagulation process, including endothelial cells, clotting factors and platelets, are activated to promote the coagulation cascade, while the increase of fibrinolytic inhibitors suppresses fibrinolysis and eventually leads to thrombosis (8–13). Therefore, sepsis-induced coagulation dysfunction is a thrombotic type (14, 15). Early thrombosis prevents the spread of pathogens and is beneficial, known as “immunothrombosis” (10, 16). As the disease progresses, widespread thrombosis will block tissue perfusion, resulting in organ dysfunction (17, 18). Some scholars considered sepsis-induce coagulopathy (SIC) as a matter of timeline (19). Autopsies of sepsis patients revealed multiple microthrombi formations in the capillaries of various vital organs including the lung, brain and kidney, indicating that widespread thrombosis led to multiple organ dysfunction and even death (20). Activated coagulation in sepsis is initiated by the extrinsic coagulation pathway. It has been reported that tissue factor (TF) monoclonal antibody inhibited sepsis-induced coagulation activation and fibrin deposition in the lung and kidney, and improved the severity of lung injury and kidney injury (21). The results of sepsis phenotypes by machine learning indicated that the phenotype with the highest mortality had the most severe organ dysfunction and the most obvious coagulation activation (22, 23). Therefore, coagulation activation plays a central initiating role in the development of MODS in sepsis, rather than one of the involved organs (2). Consequently, anticoagulant therapy has emerged as one of the most essential and promising treatment strategies for managing sepsis.

## 3 The effects of anticoagulant therapy in sepsis

Coagulation activation and thrombosis play an important role in the pathophysiological process of sepsis. Anticoagulant therapy is necessary to improve organ dysfunction and even prognosis in sepsis patients.

Bernard et al. (24) reported the role of recombinant human activated protein C (rhAPC) in patients with severe sepsis (PROWESS study) in 2001. They concluded that treatment with rhAPC was associated with an absolute 6.1% reduction in the risk of death, which highlights the potential effect of anticoagulant therapy for sepsis patients. Surviving Sepsis Campaign (SSC) guidelines 2016 (25) put forward the section on anticoagulants in sepsis, referring to the use of recombinant human thrombomodulin (rhTM) or heparin for the treatment of sepsis or septic shock. No recommendations have been made for the time being due to the lack of positive results from RCTs, indicating that anticoagulant therapy in sepsis has gained worldwide attention. The Japanese Clinical Practice Guidelines for Management of Sepsis and Septic Shock (J-SSCG) 2020 (26) recommended the administration of antithrombin (AT) and rhTM for patients with sepsis-associated DIC. This recommendation was made despite the lack of definitive evidence from large RCTs supporting the use of these therapies. Although SSC guidelines 2021 (27) do not have a dedicated section on anticoagulants, this does not necessarily mean that sepsis patients do not require anticoagulant therapy. The role of anticoagulants in the management of sepsis remains an area of ongoing research and debate. At present, there is a lack of significant results from large RCTs evaluating the use of anticoagulant therapies on sepsis patients over the past five years. Highlights the inherent complexity and challenges surrounding the potential role of anticoagulation in the management of this clinical syndrome, such as the identification of the optimal targeted patients for anticoagulant intervention, the proper timing, the selection of specific anticoagulants, and establishment of effective dosing regimens remain highly contentious and unresolved issues. Previous phase 2b and 3 multicenter RCTs on anticoagulant therapy in sepsis or septic shock, including tissue factor pathway inhibitor (TFPI) (OPTIMIST study) in 2003 (28), APC in 2012 (29), AT in 2013 (30) and rhTM in 2013 (31), explored the effects of anticoagulants in patients with sepsis or severe sepsis. All studies failed to detect any significant results with mortality rate. However, subgroup analyses of PROWESS study (24), KyberSept study (32) and RESOLVE study (33) showed that anticoagulant therapy reduced the mortality of sepsis patients with DIC, especially with overt DIC (34), suggesting that there are targeted patients for anticoagulant therapy in sepsis. Subsequently, Yamakawa et al. (35) reported that anticoagulant therapy might be associated with reduced mortality in subsets of sepsis patients diagnosed with DIC and/or very severe disease [sequential organ failure assessment (SOFA) score 13-17]. It seems that the optimal patients for anticoagulant therapy in sepsis are those with DIC and high disease severity.

Sepsis, acute respiratory distress syndrome (ARDS) and DIC are all clinical syndromes with great heterogeneity, especially DIC (36, 37). With the development of artificial intelligence (AI), Seymour et al. (22) divided sepsis patients into α, β, γ, and δ phenotypes by machine learning method in 2019, among which the mortality rate was highest in the δ phenotype. The δ phenotype was characterized by the most obvious coagulation changes, mainly manifested as increased thrombin-antithrombin (TAT), D-dimer (D-D), and plasminogen activator inhibitor-1 (PAI-1). In 2021, Kudo et al. (23) divided sepsis patients into dA, dB, dC, and dD phenotypes by machine learning method. Cluster dA had the most severe coagulopathy with high levels of D-D and fibrinogen degradation product (FDP), the most severe organ dysfunction, and the highest mortality. The therapeutic effects of rhTM varied across sepsis phenotypes, and a sepsis phenotype with high D-D and FDP may be the targeted patients of rhTM (38). These studies suggest that due to the heterogeneity of sepsis and DIC, not all the sepsis patients will benefit from anticoagulant therapy. It is necessary to apply AI to find the targeted patients of anticoagulant therapy based on relevant clinical parameters and biomarkers, which is also the focus of current research.

## 4 Clinical data evaluating the efficacy of heparin in sepsis patients

In addition to identifying the targeted patients for anticoagulant therapy in sepsis, it is particularly important to select the appropriate anticoagulant agents. Various anticoagulation agents showed different mechanisms of action in clinical practice. For example, heparin includes unfractionated heparin (UFH) and low-molecular-weight heparin (LMWH). The molecular weight of UFH ranges from 6000 to 20000 Dalton. UFH mainly binds to the lysine site on AT and inhibits the action of thrombin and factor (F) Xa. UFH inhibits the effects of thrombin by up to 1000 times (39). LMWH, with a molecular weight of 3000-7000 Dalton, acts by inhibiting FXa. Therefore, UFH has more biological activities than LMWH. As early as 2006, Zhang et al. (40) from our team reported the therapeutic effects of early administration of low-dose heparin in patients with severe sepsis, and results showed that early administration of UFH improved coagulation function and shortened the length of intensive care unit (ICU) stay in patients with severe sepsis. This is the first RCT reported to evaluate the effects of UFH in sepsis patients. Then in 2009, we further reported the clinical efficacy of low-dose heparin therapy in 79 sepsis patients (41). The results showed that low-dose UFH decreased the morbidity of DIC, acute renal failure (ARF) and MODS in sepsis patients. UFH also reduced the 28-day mortality rate without severe side effects. These two early RCTs opened the way to investigating the effects of UFH on sepsis patients.

In 2008, Zarychanski et al. (42) conducted a retrospective, propensity-matched, multicenter cohort study of 2356 patients diagnosed with septic shock, of which 722 received intravenous UFH. The study concluded that early intravenous UFH was associated with decreased mortality when administered to patients diagnosed with septic shock, especially in patients with higher severity of illness. Subsequently, Jaimes et al. (43) performed a randomized, double-masked, placebo-controlled, single-center clinical trial (the HETRASE Study) in 2009 to evaluate the effects of UFH on sepsis patients. Unfortunately, the study failed to prove a beneficial effect on acute physiology and chronic health evaluation (APACHE) II score, length of hospital stay and the mortality rate. The results of these clinical studies have been inconsistent. It might be related to the differences in the severity of sepsis patients, combined with DIC or not, the usage and dosage of UFH, and the primary outcomes. Therefore, in 2014, our team conducted a systematic review of heparin in the treatment of sepsis (44), including 1167 patients with sepsis from 17 RCTs. The results showed that heparin reduced the severity and mortality rate of sepsis without noticeable adverse effects. Another meta-analysis of heparin therapy in patients with severe sepsis reported by Wang et al. (45) in 2014 included 9 studies with a total of 3482 sepsis patients. The results showed that heparin decreased 28-day mortality in patients with severe sepsis (30.36% in the heparin group vs. 38.04% in the control group) without increasing the risk of bleeding. In a 2015 meta-analysis by Zarychanski et al. (46), a total of 2637 sepsis patients from 9 RCTs were included. The results indicated that heparin in patients with sepsis, septic shock, and sepsis-induced DIC might be associated with a 12% relative risk reduction in mortality. All three meta-analyses reached a consistent conclusion that heparin reduced the mortality of sepsis patients. However, the heparin used in the above studies included both UFH and LMWH, which have different pharmacological effects. In the meta-analysis by Wang et al. (45), 40% of the study group patients used UFH exclusively, compared to 11% in Zarychanski et al’s study (46). Furthermore, in Wang et al’s meta-analysis, non-RCTs accounted for most of the weight on mortality, while Zarychanski et al’s study included only RCTs, but the time period ranged from 1983 to 2014. These differences may account for the difference in mortality reduction between the two meta-analyses. Sepsis is a clinical syndrome involving multiple systems such as inflammation, coagulation and immunity. Therefore, UFH, which has multiple biological effects, may be a more suitable choice (47). Based on these results, in 2022, our team performed a meta-analysis of the clinical efficacy of UFH in sepsis patients (48). We included 2617 patients from 15 RCTs. The meta-analysis suggested that UFH might decrease 28-day mortality and improve the clinical efficacy, including lower MODS incidence, length of ICU stay and ventilation time in sepsis patients without bleeding complications. This further supports the use of UFH in sepsis patients.

With the development of AI, Peng et al. (49) conducted a retrospective analysis of the Medical Information Mart for Intensive Care (MIMIC)-III database to explore the efficacy of UFH in SIC patients. A total of 1820 SIC patients were included. The results showed that anticoagulant therapy with UFH was associated with reduced 28-day mortality and hospital mortality in SIC patients without increasing the risk of intracranial hemorrhage or gastrointestinal bleeding. In the same year, Zou et al. (50) examined the effect of early prophylactic anticoagulation with heparin in sepsis patients based on MIMIC-IV database. A total of 6646 sepsis patients were included, and the results showed that heparin significantly reduced in-hospital mortality. In fact, the subgroup analyses of heparin effects in sepsis from several previous large RCTs of anticoagulants including APC (PROWESS) (20), AT (KyberSept) (32) and rhTFPI (OPTIMIST) (28) were evaluated. The control patients in each study were divided into heparin group and non-heparin group. The results showed that heparin alone could significantly improve the 28-day survival rate in all three studies (51). The 28-day survival rate in the heparin and non-heparin groups were 71.9% vs. 60.6% in PROWESS study (*P*=0.002), 63.5% vs. 56.5% in KyberSept study (*P*=0.03), and 71.5% *vs.* 58.9% in OPTIMIST study (*P*<0.001). Therefore, at present, several small RCTs, subgroup analyses of large RCTs, meta-analyses, and reports based on MIMIC databases all support the use of heparin, especially UFH, in sepsis patients (**Table 1**). It fully demonstrated the prospect of heparin in sepsis patients.

**Table 1:** The clinical data evaluating the efficacy of heparin in sepsis patients.

References	Year	Inclusion criteria	*n*	Design	Therapy	Primary outcome	Heparin	Control	*P* Value
Warren et al. (34) (KyberSept)	2001	Severe sepsis (sepsis 1.0)	2314/1155 in control group	Phase 3 RCT	Prophylactic treatment with UFH or LMWH (≤ 10 000 IU subcutaneous per day), and heparin flushes (IV of ≤ 2 IU/kg per hour)	28-day mortality	Control subgroup 38.7%		0.94
							**Heparin**	**Non-heparin**	
							36.6%	43.6%	
Bernard et al. (26) (PROWESS)	2001	Severe sepsis (sepsis 1.0)	1690/840 in control group	Phase 3 RCT	Prophylactic treatment with a dose of UFH of up to 15,000 U per day	28-day mortality	Control subgroup		0.005
							**Heparin**	**Non-heparin**
							28%	39%
Abraham et al. (30) (OPTIMIST)	2003	Severe sepsis (sepsis 1.0)	1754/992 in control group	Phase 3 RCT	At least 1 dose of UFH or LMWH	28-day mortality	Control subgroup		0.88
							**Heparin**	**Non-heparin**
							29.8%	42.7%
Zhang et al. (42)	2006	Severe sepsis (sepsis 1.0)	22	RCT	Intravenous UFH 3‐4 IU/kg per hour	Cure rate	81.8%	54.5%	<0.05
Zarychanski et al. (44)	2008	Septic shock (sepsis 1.0)	695	Propensity- matched	Intravenous heparin within 48 h of ICU admission	28-day mortality	40.1%	44.2%	0.05
Jaimes et al. (45) (HETRASE)	2009	Sepsis (sepsis 1.0)	319	RCT	Heparin 500 U/ h × 7 d	LOS	12 d (median)	12.5 d (median)	0.976
Zhao et al. (43)	2009	Sepsis (sepsis 1.0)	79	RCT	Intravenous UFH 40‐50 mg per day	28-day mortality	15.4%	32.4%	0.03
Liu et al. (46)	2014	Sepsis (sepsis 1.0)	1167	Meta-analysis	UFH or LMWH	28-day mortality	OR = 0.59 95%CI [0.45, 0.77]		0.0001
Wang et al. (47)	2014	Sepsis (sepsis 1.0, 2.0)	3482	Meta-analysis	UFH or LMWH	28-day mortality	30.36%	38.04%	<0.0001
Zarychanski et al. (48)	2015	Sepsis (sepsis 1.0)	2637	Meta-analysis	UFH or LMWH	Mortality	RR = 0.88 95%CI [0.77, 1.00]		
Fu et al. (50)	2022	Sepsis (sepsis 1.0, 2.0, 3.0)	2617	Meta-analysis	UFH	28-day mortality	RR = 0.82 95%CI [0.72, 0.94]		
Peng et al. (51)	2022	Sepsis-induced coagulopathy (sepsis 3.0)	1820	Retrospective analysis	UFH	28-day mortality	16.9%	37.7%	<0.001
Zou et al. (52)	2022	Sepsis (sepsis 3.0)	6646	Retrospective cohort study	Prophylactic use of UFH or enoxaparin 5000 IU subcutaneously	In-hospital mortality	14.9%	18.3%	<0.001

*LMWH* low molecular weight heparin, *LOS* length of stay, *OR* odds ratio, *RCT* randomized controlled trial, *RR* relative risk, *UFH* unfractionated heparin.

Bold means two subgroups below the control subgroup.

Sepsis and DIC are both clinical syndromes, and the drug efficacy is greatly affected by patient heterogeneity, which may be the main reason for the lack of convincing positive results of anticoagulant therapy in sepsis over the years. However, with the development of AI, an increasing number of studies based on AI suggests that there may be different phenotypes of sepsis and that not all sepsis patients benefit from anticoagulant therapy (22, 23, 38). AI can analyze large amounts of clinical data to support clinical diagnosis and decision-making earlier and more accurately than traditional methods. Therefore, it is necessary to apply AI to classify sepsis patients with different coagulopathy phenotypes and to identify the targeted subgroups. Through the continuous training and validation of AI models, it is expected to realize individualization and precision of anticoagulation therapy and improve the prognosis of sepsis patients. In 2022, Guo et al (52) identified four phenotypes (C_1, C_2, C_3, C_4) of sepsis patients in MIMIC datasets using deep learning and traditional machine learning. Patients in C_1 showed low white blood cell count, low neutrophil proportion, but the highest lymphocyte proportion. Patients in C_2 showed lowest SIC and SOFA score, and the highest survival rate. Patients in C_3 showed significantly prolonged partial thromboplastin time (PTT), high SIC score, and a higher proportion of using heparin compared to patients in other clusters. Patients in C_4 showed abnormal coagulation with slightly prolonged PTT, and the worst prognosis. The early mortality rate of patients in C_3 was higher than C_4. However, the long-term survival rate of patients in C_3 was significantly better than patients in C_4, indicating that the anticoagulation effects of heparin improved organ failure caused by extensive micro thrombosis. Furthermore, the results confirmed anticoagulant therapy is associated with reduced mortality only in subgroups of patients with SIC and/or severe illness (35). It is therefore particularly important to conduct further studies to identify the appropriate subtypes of sepsis for heparin anticoagulation.

In present clinical practice, laboratory tests play a central role in evaluating the severity of coagulopathies and the effectiveness of heparin therapy in sepsis, mainly those parameters related to the pathophysiological processes of sepsis-induced coagulation activation, such as prothrombin time (PT), international normalized ration (INR), D-D, FDP and platelet (53). In fact, these are hemostasis parameters included in the diagnosis of SIC and DIC. It is worth noting that coagulation activation in sepsis is complex, and these coagulation parameters will change dynamically over time. It is essential to monitor continuously and analyze them in conjunction with clinical course and manifestations (20). Parameters such as TAT and PAI-1 are valuable coagulation biomarkers in sepsis, and they may reflect the severity and prognosis of sepsis (54). However, they are not currently available for routine clinical use. Thus, they are not being utilized in the monitoring of the anticoagulant therapy in the management of sepsis.

In addition to proper drugs, there are still many conflicts about anticoagulant therapy for sepsis patients. First, the criteria for the effectiveness of treatment. It may not be appropriate to use the 28-day mortality rate as the primary outcome. Sepsis is a clinical syndrome with a high degree of heterogeneity. Therefore, it may be more proper to use parameters such as the improvements in organ function as the primary outcome. Second, the targeted patients of anticoagulant therapy. Many studies have evaluated the proper patients for anticoagulant therapy in sepsis, but there is still no consensus. Severe sepsis patients? SIC patients? Sepsis patients with DIC? Which diagnostic criteria for DIC? Or the targeted patients defined by AI? Further studies are needed. Third, suitable anticoagulants. Heparin is the most commonly used anticoagulant in China, while AT or rhTM is widely used in Japan. The action of different anticoagulants is distinct, so the effects in sepsis patients are also divergent. Fourth, usage and dosage. Subcutaneous or intravenous administration for heparin? Single injection or continuous pumping? The dosage? Fifth, the proper timing. The formation of immunothrombosis is beneficial for pathogen clearance in the early stage of sepsis. As the disease progresses, coagulation activation is further aggravated, leading to widespread microvascular thrombosis and tissue hypoxia, which in turn leads to organ dysfunction. There seems to be a potential therapeutic window for anticoagulant treatment in sepsis (17). However, sepsis patients commonly present to the hospital at a late stage of infection, especially elderly patients. Some patients may even have already progressed to septic shock by the time of admission and might have been in the stage of uncontrolled/dysregulated immunothrombosis. In addition, sepsis patients admitted to ICU have already been through the emergency department, the general ward or the operating room and may have passed the early stage of sepsis by the time they are transferred to ICU, even if they arrive at the hospital early. Moreover, no clinical indicators suggesting the potential therapeutic window in sepsis. Still there are many clinical and laboratory parameters indicating coagulation activation and the need for anticoagulant therapy. Accordingly, considering clinical practice, there is no idealized therapeutic window for anticoagulant therapy. All of the controversies are yet to be answered by large RCTs.

## 5 Possible mechanisms of heparin in sepsis

**Figure 1:**
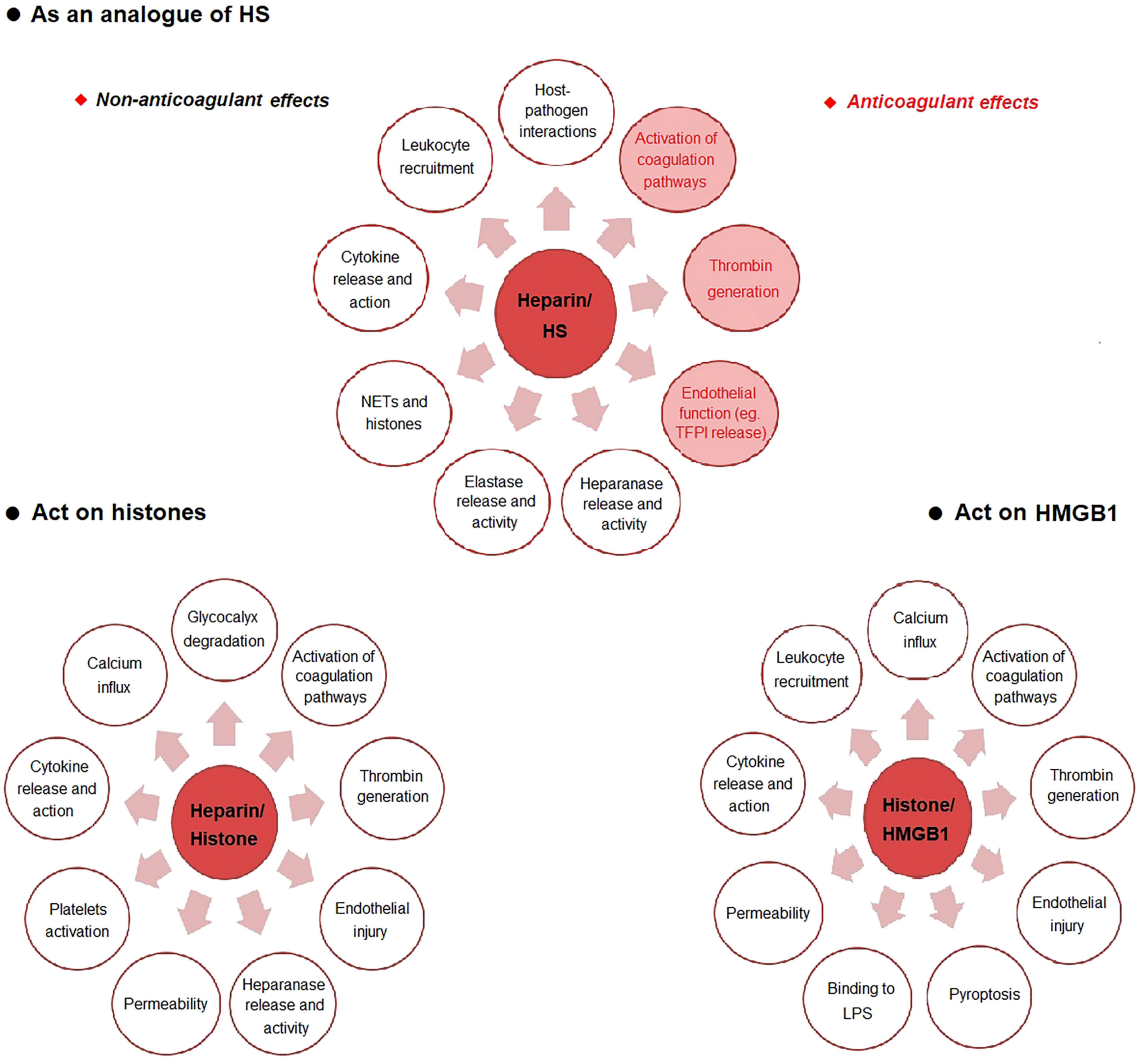
The possible mechanism of heparin in sepsis. *HS* heparan sulfate, *LPS* lipopolysaccharide, *NETs* neutrophil extracellular traps.

Recent studies have shown that damage-associated molecular patterns (DAMPs) play a crucial role in sepsis, especially HMGB1 and histones (55). These DAMPs damage a variety of cells, including endothelial cells, epithelial cells, cardiomyocytes, platelets, resulting in inflammatory response, increased vascular permeability and coagulation activation, and therefore are important mediators promoting the development of sepsis. As an anticoagulant commonly used in clinical practice, UFH has many biological activities such as anti-inflammatory, immunomodulatory effects and protecting endothelial cells (47). UFH can protect many kinds of cells, such as endothelial cells, neutrophils, platelets and so on. It also has beneficial effects on the lung, liver, kidney, coagulation system and immune system (**Figure 1**; **Table 2**).

**Table 2:** Mechanisms of heparin in sepsis.

	Mechanism of action in sepsis	References
As an analogue of heparan sulfate		
**anticoagulant effects**	inhibiting the activation of coagulation pathways	(60)
	reducing thrombin generation	
	protecting endothelial cells	(61)
	improving microcirculation	(62)
**non-anticoagulant effects**	inhibiting host-pathogen interactions	(56)
	reducing leukocyte recruitment	(63)
	decreasing cytokine release and action	(61)
	reducing endothelial permeability	(64)
	inhibiting NETs formation and histone cytotoxicity	(65)
	limiting elastase release and activity	(63)
	reducing heparinase release and activity	(66)
	protecting glycocalyx	(67)
Action on histones		
	reducing histones-induced cytokine release and action	(76)
	reducing calcium influx	(77)
	inhibiting heparinase release and activity	(66, 67)
	reducing glycocalyx degradation	(67)
	decreasing endothelial injury	(67)
	limiting activation of coagulation pathways	(66)
Action on HMGB1		
	inhibiting the HMGB1-induced inflammatory response	(72)
	inhibiting HMGB1-mediated calcium influx	(79)
	decreasing leukocyte recruitment	
	reducing cytokine release	
	inhibiting LPS binding and pyroptosis	
	limiting activation of coagulation pathways	
	reducing thrombin generation	
	inhibiting endothelial dysfunction	
	inhibiting caspase-11 activation and reducing caspase-11 dependent immune responses	(80)
Action on heparin-binding protein		
	binding and inactivating to HBP	(83)
	decreasing HBP- mediated vascular hyperpermeability	(82)

*NETs* neutrophil extracellular traps, *LPS* lipopolysaccharide, *HBP* heparin- binding protein.

### 5.1 As an analogue of heparan sulfate

The protective effect of heparin on HS is one of the main mechanisms (56). Glycocalyx is an important structure on the surface of endothelial cells (57). It is composed of glycosaminoglycans (GAG), glycoproteins and proteoglycans (57). Glycocalyx is the first to be destroyed and degraded in sepsis (58, 59). HS is the core component of the glycocalyx network structure, accounting for 90% of GAG (59). HS can be destroyed by a variety of enzymes (56). Heparin has a close structure to HS, thereby reducing the direct interaction of microbes with HS on the cell surface through competition (56). Heparin exerts anticoagulant and non-anticoagulant effects by depressing HS destruction. The anticoagulant effects include inhibiting the activation of coagulation pathways and reducing thrombin generation (60), protecting endothelial cells (61), improving microcirculation (62). The non-anticoagulant effects include inhibition of host-pathogen interactions (56), reducing leukocyte recruitment (63), decreasing cytokine release and action (61), reducing endothelial permeability (64), inhibiting neutrophil extracellular traps (NETs) formation and histone cytotoxicity (65), limiting elastase release and activity (63), reducing heparinase release and activity (66) and protecting glycocalyx (67).

### 5.2 Action on histones

Another important mechanism of heparin is the direct binding to histones to reduce histone toxicity (68). Histone-mediated inflammatory response, endothelial cell damage and thrombosis, contribute to the occurrence of MODS and even death in sepsis (69). Increased levels of histones were positively correlated with the severity of sepsis (70). Recent studies have shown that circulating histones, as DAMPs molecules, play a destructive role in sepsis in a variety of ways, including inducing calcium inflow (71), directly destroying cell integrity and resulting in cell death (72), damaging endothelial cells and increasing vascular permeability (73), binding to toll-like receptors (TLRs) on the surface of macrophages and platelets to induce the release of inflammatory factors and platelet activation and aggregation (74), promoting fibrin polymerization, inhibiting plasmin activity and thus suppressing fibrinolysis (75). Heparin has a strong affinity with histones (68). Heparin reduces histone-induced cytokine release and action (76), calcium influx (77), heparinase release and activity (66, 67), glycocalyx degradation (67), endothelial injury (67) and activation of coagulation pathways (66).

### 5.3 Action on HMGB1

As a late inflammatory mediator, HMGB1 binds to TLR4 and receptor for advanced glycation end-products (RAGE) on neutrophils and macrophages to induce NETs production and cytokine/chemokine release, playing a damaging role in sepsis (78). Heparin inhibits the binding of HMGB1/LPS complex to RAGE and promotes the formation of apoA-I/LPS complex, thereby reducing the formation of LPS/HMGB1 complex and ultimately inhibiting the HMGB1-induced inflammatory response (72). Heparin also inhibits HMGB1-mediated calcium influx, leukocyte recruitment, cytokine release, LPS binding, pyroptosis, activation of coagulation pathways, thrombin generation and endothelial dysfunction (79). In particular, Tang et al. (80) reported that heparin prevents caspase-11-dependent immune responses and lethality in sepsis, independent of anticoagulant properties. Heparin inhibits caspase-11 activation by blocking cytosolic delivery of LPS through preventing glycocalyx degradation.

### 5.4 Action on heparin-binding protein

HBP is released by neutrophils as an important granule protein in sepsis. HBP is reported to be a biomarker of sepsis and is related to the severity of septic shock and organ dysfunction (81). HBP may cause vascular hyperpermeability by binding to transforming growth factor–β receptor type 2 (TGF-β-R2) as a ligand on endothelial cells (82). HBP binds to glycosaminoglycans of the glycocalyx and results in endothelial cytoskeletal rearrangement. Heparin, which is structurally similar to glycosaminoglycans, has a strong binding capacity to HBP and leads to the inactivation of HBP (83).

## 6 Conclusions

Sepsis is a clinical syndrome in which coagulation, inflammation, immunity and other systems interact. The pathophysiology of sepsis is complex. Heparin has a variety of biological activities and thus has a broad prospect in sepsis patients. At present, clinical studies on the use of heparin in sepsis are inconclusive. Subgroup analyses of large RCTs and meta-analyses suggest efficacy, but validation by large RCTs is needed. The targeted patients, proper timing, usage and dosage of administration, and the primary outcome are key to achieving positive results. The protective mechanisms of heparin in sepsis is multi-target and preliminary results have been obtained. The exact mechanisms and the interaction still need to be further elucidated.
